# Selective Decomposition of Waste Rubber from the Shoe Industry by the Combination of Thermal Process and Mechanical Grinding

**DOI:** 10.3390/polym14051057

**Published:** 2022-03-07

**Authors:** Qiao Xiao, Changlin Cao, Liren Xiao, Longshan Bai, Huibin Cheng, Dandan Lei, Xiaoli Sun, Lingxing Zeng, Baoquan Huang, Qingrong Qian, Qinghua Chen

**Affiliations:** 1 College of Chemistry and Materials, Fujian Normal University, Fuzhou 350007, China; qsz20191121@student.fjnu.edu.cn (Q.X.); qsz20191117@student.fjnu.edu.cn (L.B.); qsx20190767@student.fjnu.edu.cn (D.L.); 2 Engineering Research Center of Polymer Green Recycling of Ministry of Education, Fujian Normal University, Fuzhou 350007, China; qbx20190099@yjs.fjnu.edu.cn (H.C.); sunxiaoli@fjnu.edu.cn (X.S.); zenglingxing@fjnu.edu.cn (L.Z.); qbh811@sina.com (B.H.); qrqian@fjnu.edu.cn (Q.Q.); cqhuar@fjnu.edu.cn (Q.C.); 3 College of Environmental Science and Engineering, Fujian Normal University, Fuzhou 350007, China; 4 Fujian Key Laboratory of Pollution Control & Resource Reuse, Fujian Normal University, Fuzhou 350007, China

**Keywords:** waste rubber, thermo-mechanical grinding, filling rates, grinding temperature

## Abstract

A major challenge in waste rubber (WR) industry is achieving a high sol fraction and high molecular weight of recycled rubber at the same time. Herein, the WR from the shoe industry was thermo-mechanically ground via the torque rheometer. The effect of grinding temperature and filling rate were systematically investigated. The particle size distribution, structure evolution, and morphology of the recycled rubber were explored by laser particle size analyzer, Fourier transform infrared spectroscopy (FTIR), sol fraction analysis, gel permeation chromatography (GPC), differential scanning calorimeter (DSC), and scanning electron microscope (SEM). The results indicate that the thermo-mechanical method could reduce the particle size of WR. Moreover, the particle size distribution of WR after being ground can be described by Rosin’s equation. The oxidation reaction occurs during thermal-mechanical grinding. With the increase of the grinding temperature and filling rate, the sol fraction of the recycled WR increases. It is also found that a high sol fraction (43.7%) and high molecular weight (35,284 g/mol) of reclaimed rubber could be achieved at 80 °C with a filling rate of 85%. Moreover, the obtained recycled rubber compound with SBR show a similar vulcanization characteristics to pure SBR. Our selective decomposition of waste rubber strategy opens up a new way for upgrading WR in shoe industry.

## 1. Introduction

The rubber industry makes remarkable contribution to the world economy, and the production of rubber from the footwear and tire industries grows annually, but it also results in a large amount of waste [1,2,3]. Despite decades of research in this field, the disposal of waste rubber remains an economic, social, and environmental issue [4]. The improper disposal of waste rubber results in a large amount of waste discarded or incinerated, which causes environmental damage and even has the risk of spreading diseases, affecting dramatically the quality of human life [5].

Waste tire and footwear rubber are usually vulcanized, which is mainly composed of complex mixtures such as inorganic fillers, activators, accelerators, pigments, and stabilizers. When substances with low molar mass are discarded randomly in the natural environment, they may permeate the soil and the groundwater over time and seriously harm human health [6,7,8,9,10]. At present, it is hard to reclaim vulcanized rubber because of its three-dimensional cross-linked structure network [11]. It is therefore of great significance to come up with more effective and environmentally friendly technologies to reclaim these waste rubbers.

Generally, the existing reclamation techniques for waste rubber (WR) include microwave [12,13,14], ultrasound [15,16], chemical modification [17,18,19,20,21], ambient grinding [22], cryogenic grinding [23,24], and biological methods [25]. However, low yield and high energy consumption technologies are not suitable for industrial applications. Compared with the above-mentioned techniques, the thermo-mechanical reclaiming of vulcanized rubber wastes has aroused public attention on account of easy procedures and high yield as well as the use of commercially available equipment.

The mechanochemical reclaiming has the advantages of better processability and mechanical properties along with the enhanced interfacial bonding between reclaiming rubber powder and rubber. The mechanochemical reclaiming is based on mechanical shearing and a strong rise of temperature, brought by an external heat source as well as the friction between the crumb rubbers. This leads to devulcanization and the formation of shorter polymer chains by the rearrangement of the polysulfide cross-links and the degradation of carbon-carbon bonds [1]. It is worth noting that thermo-mechanical methods with suitable temperature and filling rate can be applied to achieve devulcanization without the devulcanizing agent [26].

A great deal of achievements have been made in the thermomechanical reclaiming of vulcanized rubber wastes. Liu and his coworker investigated the effects of mechano-chemical degradation of the crosslinked and foamed ethylene-vinyl acetate copolymer (EVA) multicomponent and multiphase waste material in a solid state by torque rheometer, and it was found that the ruptures of chemical bonds in its net and stereo-structures are caused by external mechanical energy input by the Roller rotors of the HAAKE rheometer, and the optimum mechanochemical degradation conditions are at 120–130 °C in a Roller rotor rotation speed range of 40–60 rpm for 24 min [27]. Formela et al. investigated the reclaiming of ground tire rubber (GTR) using a corotating twin-screw extruder at different barrel temperatures (60, 120, and 180 °C). The authors observed that the increase of the barrel temperature decreases the Mooney viscosity, crosslinking density, and optimum cure time (t_90_) during the revulcanization of the reclaimed GTR [28]. Recently, Zhang et al. developed a thermal-oxidative reclamation process using a newly designed dynamic reclamation reactor, in the absence of any chemical agent, a high reclamation degree of GTR (sol fraction 66.53 wt%) was obtained at 200 °C for 20 min. The authors concluded that oxidative reclamation induced cross-link breakage and main-chain scission simultaneously, destroying the cross-linked network of the GTR, which leads to the increase of the sol fraction and decrease of the molecular weight [29]. However, such thermal-mechanical recycle methods remain a great challenge in obtaining recycling rubber with both high molecular weight and sol content.

The pressure and grinding temperature play an important role in the grinding of the largest particles for WR. Increasing the pressure or reducing the grinding temperature would reduce the size of the largest particles by the combined breakage mechanism including abrasion, cleavage, and fracture [30]. However, it has been recognized that the unsaturated double bonds of the main chains combined with oxygen can cause a different degree of oxidative degradation under the action of heat [31]. Therefore, it is urgent to investigate the combination of thermal process and mechanochemical grinding.

In this work, the WR from the shoe industry was ground and reclaimed by torque rheometer, and the effects of different filling rate and grinding temperature on the structure of WR were investigated. In addition, the structure evolution and morphology of WR were also investigated in detail by using laser particle size analyzer Fourier transform infrared spectroscopy (FTIR), sol fraction analysis, gel permeation chromatography (GPC), differential scanning calorimeter (DSC), and scanning electron microscope (SEM).

## 2. Materials and Methods

### 2.1. Materials

The waste rubber from the shoe industry was collected from Huafeng Shoes Co., Ltd. (Jinjiang, China). Xylene and tetrahydrofuran (THF), analytical reagents, were purchased from Sinopharm Chemical Reagent Co., Ltd. (Shanghai, China). Styrene−butadiene rubber (SBR-1502) was purchased form Jilin Petrochemical Company (Jilin, China). Rubber ingredients, including carbon black (CB), stearic acid, zinc oxide, accelerator CBS (*N*-cyclohexyl benzothiazole-2-sulphenamide), and sulfur were of commercial grades.

### 2.2. Preparation Method

**Scheme 1:**
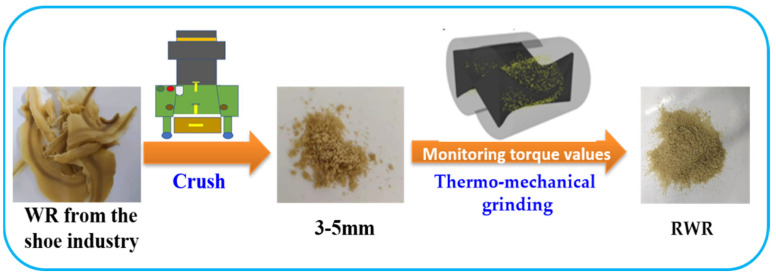
Diagram of the reclaimed waste rubber.

The waste rubber from the shoe industry was cleaned and dried, then crushed in a crusher into particles (the diameter is 3–5 mm). Then WR were ground in torque rheometer (RM200, HaPu, Haerbin, China) with two roller rotors under the condition of different temperatures and different filling rates. The speed of rotor was 40 rpm, and grinding time was 10 min, continuously measuring the rheological torque (M) of the sample (WRT/WRn, T is the grinding temperature, °C; n is the filling rate, %; for example, WR30 °C is the WR obtained at the grinding temperature of 30 °C; WR70% indicates that the filling rate is 70%) at different temperatures and different filling rates. The untreated WR was used as a control sample. The schematic diagram of the reclaimed waste rubber from the shoe industry is shown in Scheme 1.

### 2.3. Formulations and Preparation of the RWR/SBR Compound

The reclaimed waste rubber (RWR) after grinding and styrene−butadiene rubber (SBR) were composited in different formulations according to Table 1, using a two-roll mill (ZG-80T, Zhenggong Electromechanical Equipment Technology Co., Ltd., Dongguan, China) at 60 °C. RWR was first masticated with SBR for 3 min, followed by mixing with the activating agents (stearic acid and zinc oxide) for 2 min, accelerator CBS was then added to form a masterbatch, and sulfur was added finally. The samples were vulcanized at 160 °C in a flat vulcanizer.

### 2.4. Characterization

#### 2.4.1. Particle Size Distribution and Morphology

The particle size distribution of rubber powder under different conditions was measured by laser particle size analyzer (Omec Topsizer, Zhuhai, China). Scanning electron microscope (SEM, SU8100, Hitachi High-Tech, Tokyo, Japan) was used to observed the morphology of WR under different conditions. The surface of WR was vacuum plated with a thin layer of gold for electrical conduction and then observed at 5 kV.

#### 2.4.2. FTIR Test

Fourier transform infrared spectroscopy (FTIR, Nicolet IS10, Waltham, MA, USA) was used to analyze the structure and functional group changes of WR before and after treatment in the spectral range of 400–4000 cm^−1^ and in a reflection mode, with a resolution of 8 cm^−1^ and 64 times of scanning

#### 2.4.3. Raman Spectroscopy

The Raman scattering experiments were performed using a Thermo Scientific-DXR2xi spectrometer (Waltham, MA, USA). The laser is 532 nm argon ion laser, the laser power is 9 mW, and the exposure time is 0.02 s. The laser beam was focused by an ×50 magnification objective of a confocal microscope (Lycar).

#### 2.4.4. XPS Spectra

The X-ray photoelectron spectra (XPS) of the WR were recorded by using an ESCALAB Xi + spectrometer employing a monochromated Al K α X-ray source to obtain the information of elements variation.

#### 2.4.5. Thermogravimetric (TG) Test

The thermal stabilities and filler ratio of the samples were measured by a thermogravimetric analyzer (Q50, TA Instruments, New Castle, PA, USA) at a heating rate of 10 °C/min from room temperature to 800 °C. Nitrogen flow was used as the protection atmosphere with a flow rate of 20 mL/min. Approximately 10 mg of each sample was used.

#### 2.4.6. Sol-Gel Content Analysis

The sol fractions of WR were measured with a Soxhlet extraction method using xylene as the solvent. The samples (ca 0.5 g) were accurately weighed (M) and placed in a copper net, after extraction at 155 °C for 12 h, the extracted sol xylene solution was distilled below 120 °C under vacuum to become a condensed xylene solution, and then dried at 80 °C in vacuum drying chamber to a constant weight for 12 h. The gel content (M_g_) was weighted, and then the gel fraction (ω_g_) was calculated from the weight of the specimen before and after extraction with the following Equation (1). Therefore, average ω_g_ values of three times of extraction were used for each sample under different conditions. The sol fractions (ω_s_) were calculated according to Equation (2) [27].

(1) $${\;\omega }_{g}=\frac{M_{g}}{\left(1-\mathrm{filler}\;\mathrm{ratio}\right)M}\times 100\%$$

(2) $$\omega_{s}=1-\omega_{g}$$

#### 2.4.7. Molecular Weight Test

The number average molecular weight (Mn) and weight average molecular weight (Mw) of the sol samples were characterized by gel permeation chromatography (GPC, 515, Waters, Milford, MA, USA). Tetrahydrofuran was used as the solvent at ambient temperature. The polystyrene standard samples with narrow-molecular weight distribution were used as reference.

#### 2.4.8. Differential Scanning Calorimeter (DSC) Analysis

The glass transition temperature was measured by a differential scanning calorimeter (DSC, Q20, TA Instruments, New Castle, PA, USA). The samples were weighed 5~10 mg and placed in aluminum crucible. In nitrogen atmosphere, the sample was cooled to −70 °C and then heated to 200 °C at a heating rate of 10 °C/min. The corrected enthalpy (*Cp_n_*) and the weight fraction (*χ_im_*) of the immobilized polymer layer were determined by the following equations [32]:

(3) $$\Delta Cp_{n}=\frac{\Delta Cp}{1-W}$$

(4) $$\chi_{im}=\frac{\Delta Cp_{0}-\Delta Cp_{n}}{1-\Delta Cp_{0}}\;$$

where ∆*Cp* is the thermal enthalpy transition of the WR in the glass transition region, *W* is the weight fraction of the filler, and ∆*Cp*_0_ is the heat capacity transition of the untreated WR in the glass transition region.

#### 2.4.9. Vulcanization Characteristic Parameter Test

The moving die rheometer (UR-2030SD, Taiwan U-CAN Technology Co. Ltd., New Taipei City, China) was used to test the vulcanization characteristics of the rubber compound as per the standard ASTMD5289-2007a. About 5 g sample was placed between two dies and the vulcanization temperature was set to 160 °C.

#### 2.4.10. Mechanical Properties

The mechanical properties of the samples were tested in accordance with ISO 37 by a universal testing machine (CMT4104, Nss Laboratory Equipment Co., Ltd., Shenzhen, China) at a speed of 50 mm/min. Hardness was tested based on ASTM D 2240 using a Shore A durometer.

## 3. Results

### 3.1. Analysis of Waste Rubber

In order to reveal the composition and filler content of WR, the structural analysis of WR is shown in Figure S1. Combining the result of FTIR spectroscopy, Raman spectroscopy, and TG, obviously, the components of WR contain SBR and BR, and the fillers contain CaCO_3_ and SiO_2_ (white carbon—black).

### 3.2. Effect of Temperature

#### 3.2.1. Analysis of Grinding Process

**Figure 1:**
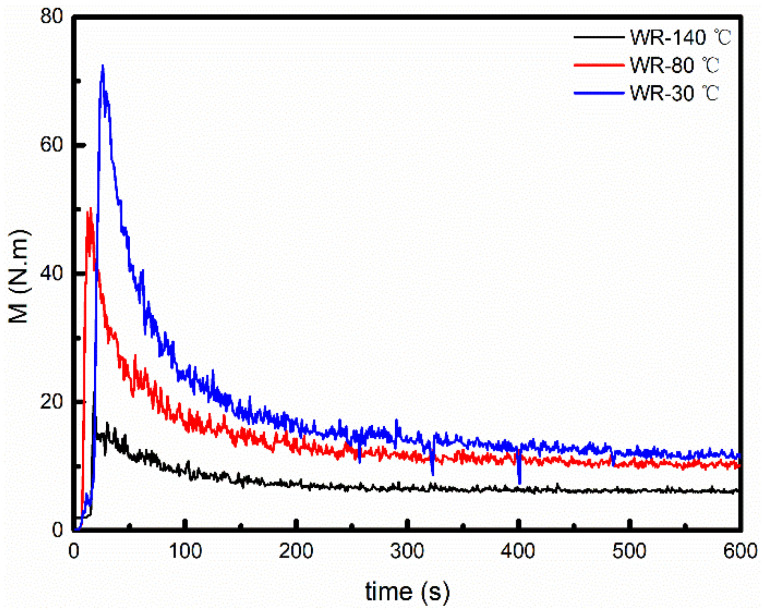
Torque (M) of the WR samples as a function of the grinding time at different temperature.

Torque rheology has been developed to an effective tool in evaluation of the grinding effect and energy consumption during mechanical recycling [2]. Torque rheology can satisfy the high need of shear force thermo-mechanical grinding. More importantly, the torque values related to the resistance of the WR face (viscosity) can be measured during grinding, which can evaluate the grinding effect. Thus, the grinding effect and energy consumption of the samples at different temperatures were analyzed by the method of torque rheology. Figure 1 is the variation of torque-time (M-t) curves for WR at different temperature. As shown in Figure 1, the torque increases rapidly at first, and then decreases as the grinding time increases, owing to bigger fragments are broken into smaller ones. With time elapsing, fragments with incomplete three-dimensional structures are continually ground, indicating the mechanical energy input by roller rotor may cause the grinding of the samples. After grinding for 6~7 min, the torque values tend to balance and fluctuate in the range of 8~15 N·m. Meanwhile, the torque of the sample decreases with the increase of grinding temperature. It is well-known that the torque value is mainly related to the viscosity of the material. The torque curves of WR and reclaimed WR at room temperature in Figure S2. Obviously, the reclaimed WR has a relatively lower torque as compared to WR, indicating lower viscosity. The damage of crosslinking part and the slip of relatively independent macromolecular chains increase the intermolecular interaction in the material, promoting the interaction friction and motion of macromolecular chains, and significantly improving the grinding effect of the material.

During the mechanical grinding in the torque rheometer, although both thermal and mechanical energy generated by the device are introduced into the samples, thermal energy can be determined from the power consumption of the device, and some heat is emitted to the surrounding environment (depending on the device surface and ambient temperature, etc.,). In addition, the friction heat generated by the friction between the sample and the rotor during the grinding process is carried away by the external cooling system. However, the mechanical energy provided by the system is measurable and can be obtained through the torque.

Therefore, grinding caused by mechanical energy was solely discussed in this paper. The relationship between power and torque can be defined as [33]:

(5) $$P=\omega M=\frac{2\pi nM}{60}=\frac{\pi nM}{30}$$

where: *ω* is angular speed, *n* is rotational speed, *M* is torque, so

(6) $$\frac{\mathrm{dE}}{\mathrm{dt}}=\frac{\pi nM}{30}$$

and

(7) $$\mathrm{dE}=\frac{\pi nM}{30}\mathrm{dt}$$

The speed *n* is constant, so the integral of both sides of the above equation can be obtained:

(8) $$Em=\frac{\pi n}{30}\int M\mathrm{dt}=\frac{\pi n}{30}M_{T}$$

where *M_T_* is the total torque, which can be obtained by system integration.

Therefore, the above formula can be used to determine the energy consumption of samples under different temperature conditions, and the results are shown in Table 2. The energy consumption of the sample decreases with the increase of grinding temperature, indicating that the increase of temperature can effectively reduce energy consumption.

#### 3.2.2. Particle Size Distribution and Morphology

**Figure 2:**
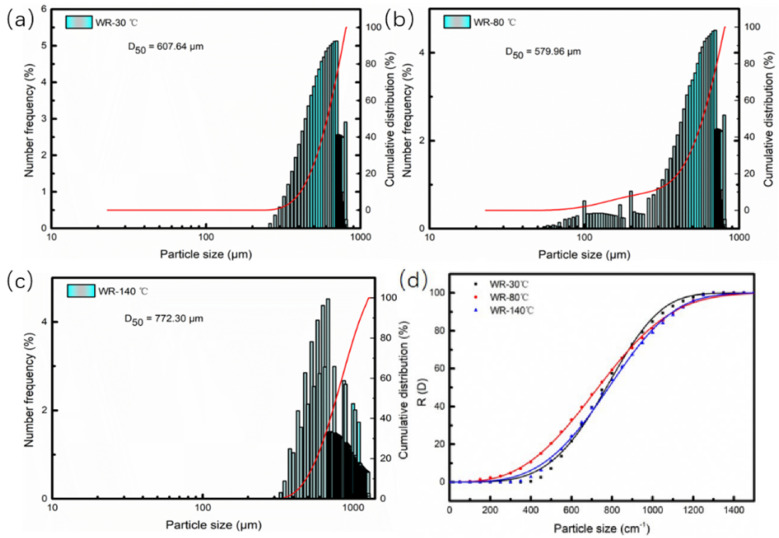
Particle size distribution of WR at different temperatures (**a**) at 30 °C, (**b**) at 80 °C, (**c**) at 140 °C, (**d**) Rosin Rammler Bennet diagram.

Figure 2 and Table S1 shows the particle size distribution of waste rubber. The grinding by torque rheometer produced smaller rubber particles with narrow distribution, the main WR particle size ranged between 500 and 1000 μm. The average particle sizes (D_50_) of WR-30 °C, WR-80 °C, and WR-140 °C are 607.64, 579.96, and 772.30 μm, respectively, demonstrating that the optimal grinding temperature is 80 °C. It should be noted that fragmentation and agglomeration of rubber particle occurred simultaneously during the grinding process. Increasing the grinding temperature to 140 °C, the rubber particles aggregate into 772.30 μm. This agglomeration is probably related to the partial devulcanization of the particles surface, which produces a sticky surface and thus the particles adhere to each other.

Rosin et al. proposed using exponential function to analyze particle size distribution function of pulverized materials in powder engineering on the basis of probability and statistics theory, as follows [34]:

(9) $$F\left(D\right)=\frac{d\varphi }{dD}=100nbD^{n-1}\exp\left(-bD^{n}\right)$$

where *b* and *n* are constants, and the *n* value is related to the width of the particle size distribution. The wider the distribution, the smaller the value. *φ* is the mass percentage of particles with particle size less than *D*. In contrast, the mass percentage of particles larger than *D* can be expressed by *R*, as follows:

(10) $$R\left(D\right)=\varphi =100-100\exp\left(-bD^{n}\right)$$

In Equation (8), *b* can be expressed as *b* = $\frac{1}{D_{e}^{n}}$, thus

(11) $$R\left(D\right)=100-100\exp\left[-{\left(\frac{D}{D^{e}}\right)}^{n}\right]\;$$

Equation (11) is applied to the experimental data set, and Origin 9 software is used to fit the experimental data. Figure 2 is the fitting relation diagram of particle size and mass percentage, and Table 3 lists the *D^e^*, R^2^, and *n* values obtained by fitting.

R^2^ coefficient determines the minimum sum of squares of residuals between experimental data and model data, and it can estimate the regression standard error between experimental data and fitting model. The coefficient can be described by the following formula [35]:

(12) $$R^{2}=1-\frac{RSS}{TSS}$$

where $RSS=\sum_{i}^{n}\;{\left(D_{ical}-D_{iexp}\right)}^{2}$ represents the sum of residual squares. $TSS=\sum_{i}^{n}\;{\left(D_{ical}-D_{aver}\right)}^{2}$ represents the sum of total squares. *D_iacl_*, *D_iexp_*, *D_aver_* are the calculated value, experimental value, and experimental average value respectively.

The results show that when the grinding temperature is 80 °C, the characteristic particle size De and its uniformity coefficient N value of WR are the minimum (Table 3). It shows that the particle size of powder decreases and the particle size distribution becomes wider in the milling process. In addition, the fitting model shows good correlation with R^2^ coefficients (>99%), indicating that the particle size distribution of WR can be analyzed by Rosin’s equation.

**Figure 3:**
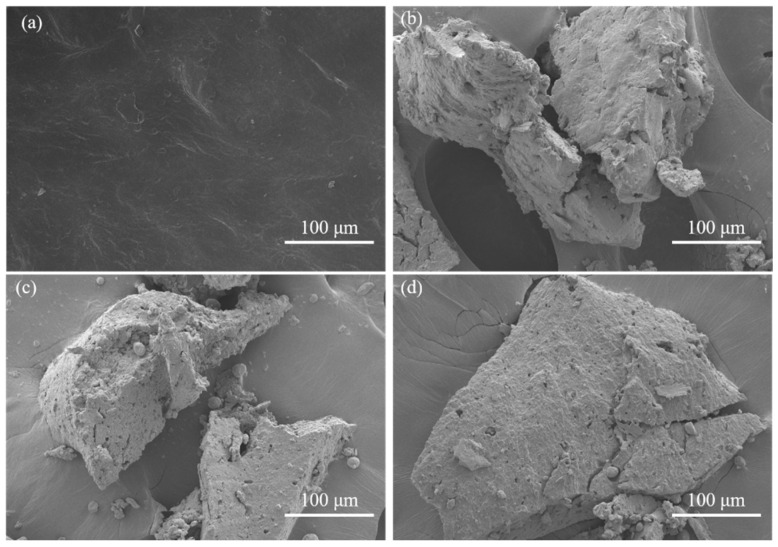
SEM images of different temperature fractured surface of WR: (**a**) untreated; (**b**) at 30 °C; (**c**) at 80 °C; and (**d**) at 140 °C.

The morphology of WR can be observed by SEM, as displayed in Figure 3. It is found that the WR without treatment has a smooth surface. After grinding, irregular particles with rough surface were formed. The morphology of WR particles ground at 30 °C has litter cracks on the surface in comparison with WR particles ground at 80 and 140 °C. With the increase of the grinding temperature, rubber particles become coarser with many smaller particles on the surface. The cross-linked rubber undergoes strong pressure and shear force during the grinding process. The inhomogeneity of the network could generate stress concentration, resulting in the fracture of some network chains.

#### 3.2.3. FTIR and Elemental Analysis

**Figure 4:**
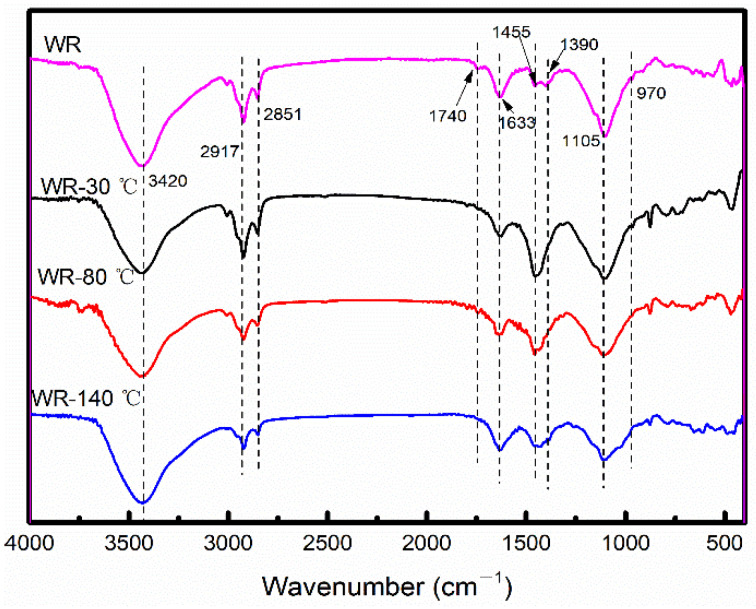
FT-IR spectra of WR at different temperature.

The infrared spectrum of WR after grinding at different temperatures is shown in Figure 4. Strangely, the absorption peaks of the reclaimed WR at different temperatures show no obvious changes, probably indicating that the main chain structure of the rubber molecular chain has no significant changes during the thermal-mechanical grinding. To further understand this process, XPS test was performed in the following part.

The elemental analysis of WR and reclaimed WR were tested by XPS spectra, as shown in Table 4. The content of O1s is only 2.26 wt%. While this value is up to 12.62 wt% after being ground at 80 °C, and 9.21 wt% for 140 °C. Obviously, the oxidation reaction occurred during the thermal-mechanical grinding, originating from some rubber molecular chains reacting with oxygen under the conditions of heat, oxygen, and mechanical force [27].

#### 3.2.4. Sol Fraction and Molecular Weight Analysis

**Figure 5:**
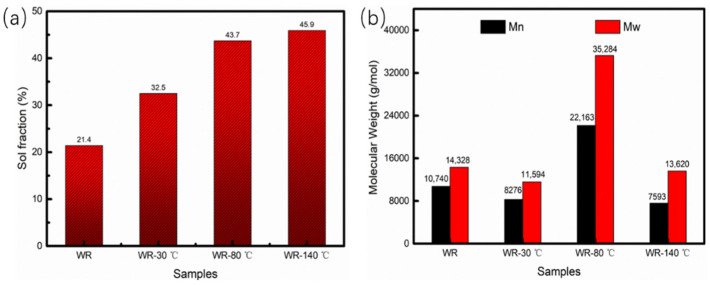
(**a**) Sol fraction of WR as a function of different temperature. (**b**) Molecular weight of WR at different temperature.

Soxhlet extraction method was used to measure the sol fraction of WR at different temperatures to evaluate the effectiveness of crosslinked structure failure during the grinding process. Figure 5a shows that thermal-mechanical grinding has a significant effect on the gel fraction of WR. The sol fraction of the original WR is 21.4%. It is obvious that the sol fraction related to the crosslinking density of materials increases significantly with the increase of temperature, from 32.5% (30 °C), 43.7% (80 °C) to 45.9% (80 °C), indicating that the crosslinking structure of WR is significantly damaged when the temperature increases.

The roller rotor of the torque rheometer used in the experiment can exert quite strong normal force and shearing force to the WR material. In addition, the crosslinking point of the WR also cause the prestressed chain to move close enough to become crosslinked, which limits the movement of the molecular chain. Hence, the molecular network structure is more likely to move during the grinding, while the crosslinked structure of WR material more easily destroys the molecular chain because of its high stress and torque. The measurement of sol fraction is positive evidence that the crosslinked structure of WR is mainly undermined by thermo-mechanical force.

Soluble components in WR before and after thermal mechanical grinding were characterized by GPC. Figure 5b shows the molecular weight measurements of WR after grinding at different temperatures. It is shown that the soluble parts of different samples have different molecular weight distributions. Compared with untreated WR, the weight average molecular weight (Mw) and number average molecular weight (Mn) of WR were the highest at 80 °C, which may be associated with the fact that the crosslinking structure of WR was effectively destroyed in the grinding process. Additionally, the decrease of Mw and Mn at 30 °C and 140 °C indicates that the main chain was more seriously damaged except for the crosslinked structure.

Due to the existence of crosslinked structure, there are almost no soluble rubber molecules in the soluble components of the original WR material. However, due to the combination of mechanical forces and temperature, there are a large number of soluble rubber molecules in the soluble components of WR after grinding, resulting in a variation in the molecular weight of the material. According to the results, it can be concluded that the crosslinked structure was obviously selectively destroyed after thermo-mechanical grinding at 80 °C, resulting in the increase of molecular weight. Since the molecular weight of the soluble portion of WR increased after grinding, the significant reduction of the gel portion may be attributed to the destruction of the crosslinked structure and the main chain, which leads to the molecular separation and weight loss.

#### 3.2.5. Analysis of DSC

**Figure 6:**
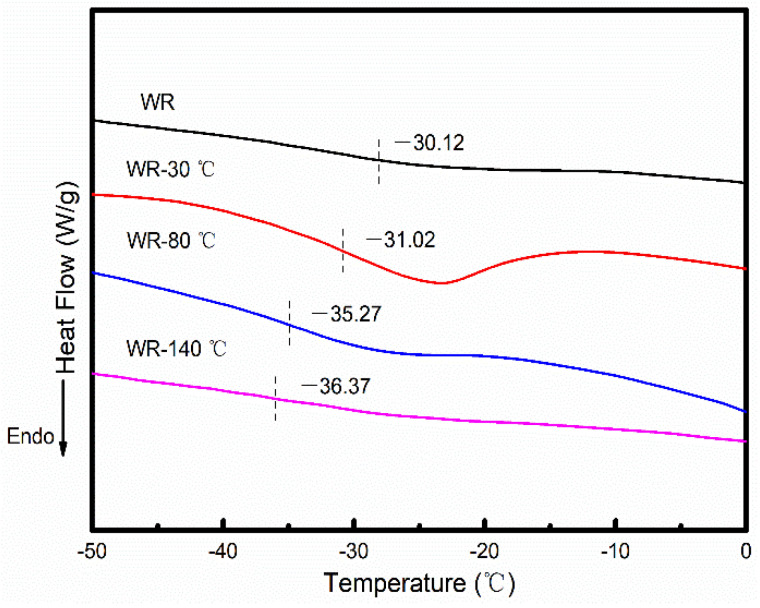
DSC curves of WR at different temperature.

The DSC thermograms of WR are presented in Figure 6, and the related values are shown in Table S2. A single glass transition temperature (T_g_) at around −30.12 °C–36.37 °C is observed for WR after various grinding temperatures. The value of T_g_ and ∆*Cp* for the WR without treatment is the highest, demonstrating that the motion of molecular chains were limited by the cross-linked networks with the maximum enthalpy [32]. With the temperature increasing, these values gradually decrease as the motion of molecular chains improves. Moreover, the values of *χ_im_* increasing trend, indicating a decrease in the fraction of immobilized polymer chains, which is in good agreement with the obtained result from sol fraction [36].

### 3.3. Effect of Filling Rate

#### 3.3.1. Analysis of Grinding Process

**Figure 7:**
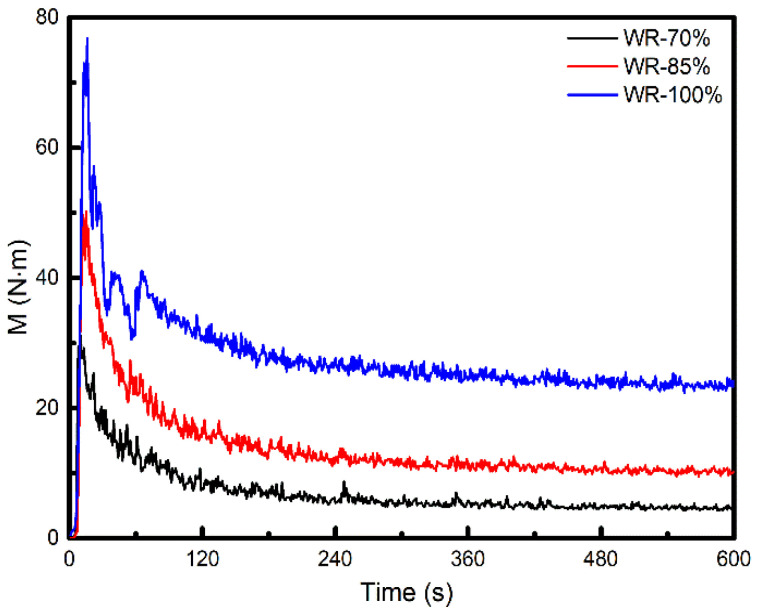
Torque (M) of the WR samples as a function of the grinding time at different filling rate.

Figure 7 shows the variation of WR torque with time at different filling rates. As shown in Figure 6, the torque of WR increases with the increase of filling rate after thermo- mechanical grinding. At the beginning, the rapid increase of torque was caused by the high pressure and shear force generated between the roller rotor, the internal mixing, and grinding chamber causing the sheet-shaped WR to be quickly broken into particle fragments, and then the torque value is stabilized, because the torque value is mainly related to the resistance the WR faced.

The energy consumption at different filling rates can be calculated by Formula (6), and the results are shown in Table 5. The energy consumption of WR increases with the increase of the filling rate, indicating that the enlargement of filling rate obviously increases the energy consumption.

#### 3.3.2. Particle Size Distribution and Morphology

**Figure 8:**
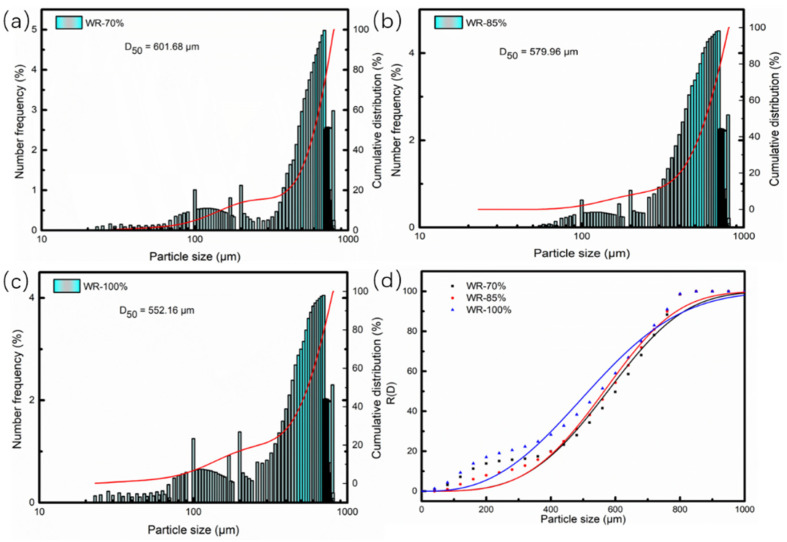
Particle size of WR at different filling rates. (**a**) at 70%, (**b**) at 85%, (**c**) at 100%, (**d**) Rosin Rammler Bennet diagram.

The particle size distribution curves of WR at different filling rates are shown in Figure 8 and Table S3. The higher the filling rate, the smaller the average particle size, indicating that the effective crushing of WR can be achieved after increasing the filling rates. The above result can be interpreted by the increase of stored elastic energy caused by the increase of the WR pressure.

Figure 8d shows the Rosin diagram of WR after grinding at different filling rates. R (D) of the powder after milling has a favorable correlation with D, suggesting that the particle size distribution of WR can be analyzed by the equation proposed by Rosin. Table 6 shows the values of De, R^2^, and N obtained through fitting. The results show that, when the filling rate increases, the characteristic particle size De of WR decreases and its uniformity coefficient N also changes, indicating that the group particle size of WR presents a downward trend.

**Figure 9:**
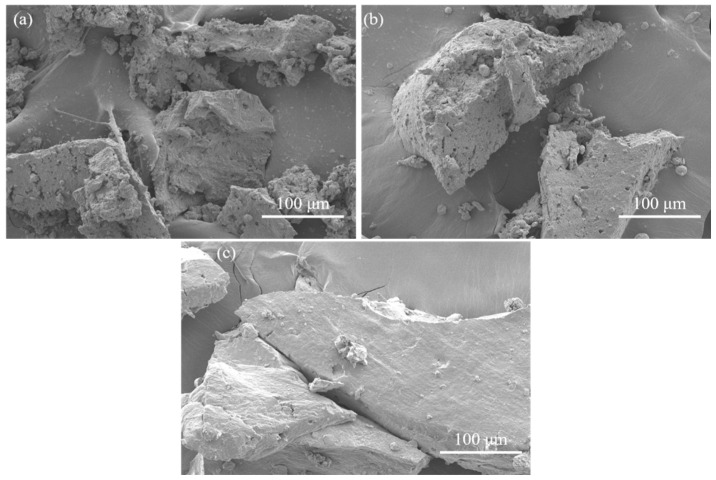
SEM images of different filling rate fractured surface of WR: (**a**) at 70%; (**b**) at 85%; and (**c**) at 100%.

The micrographs of different filling rate fractured surface of WR are shown in Figure 9. When the filling rate was 70%, the sample shows a fairly rough surface, presenting ductile behavior. The increase of filling rate is accompanied by the increase of pressure. Under the strong pressure, the main molecular chain or the crosslinking bonds of WR are instantly destroyed, thus being decomposed into smooth irregular particles. It is obviously found that the obtained sample shows a relatively smoother surface as the filling rate increases to 100%, indicating brittle failures during the grinding.

#### 3.3.3. Analysis of FTIR

**Figure 10:**
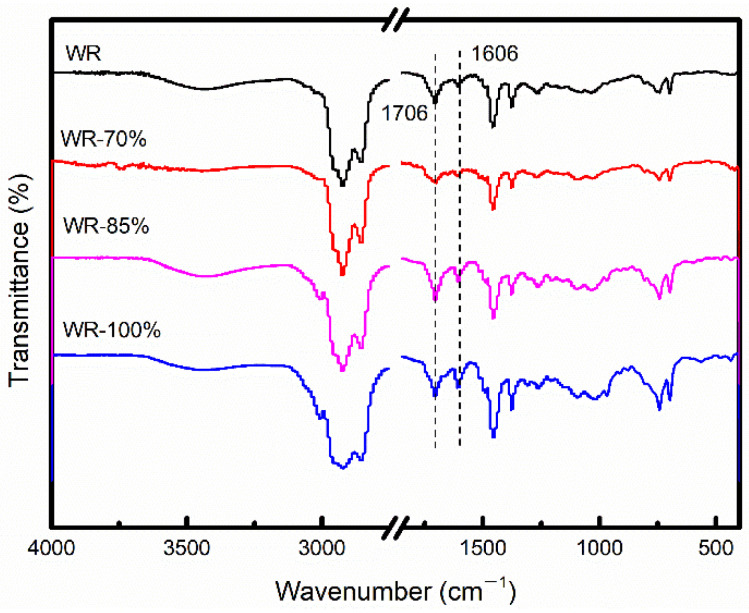
FTIR spectra of WR at different filling rate.

The infrared spectrum of WR after grinding at different filling rates is shown in Figure 10. It can be seen from the figure that the absorption peak essentially unchanged with the increase of filling rate, explaining that the enlargement of pressure will not destroy the main chain structure of WR and protect the integrity of the molecular chain.

#### 3.3.4. Sol Fraction and Molecular Weight Analysis

**Figure 11:**
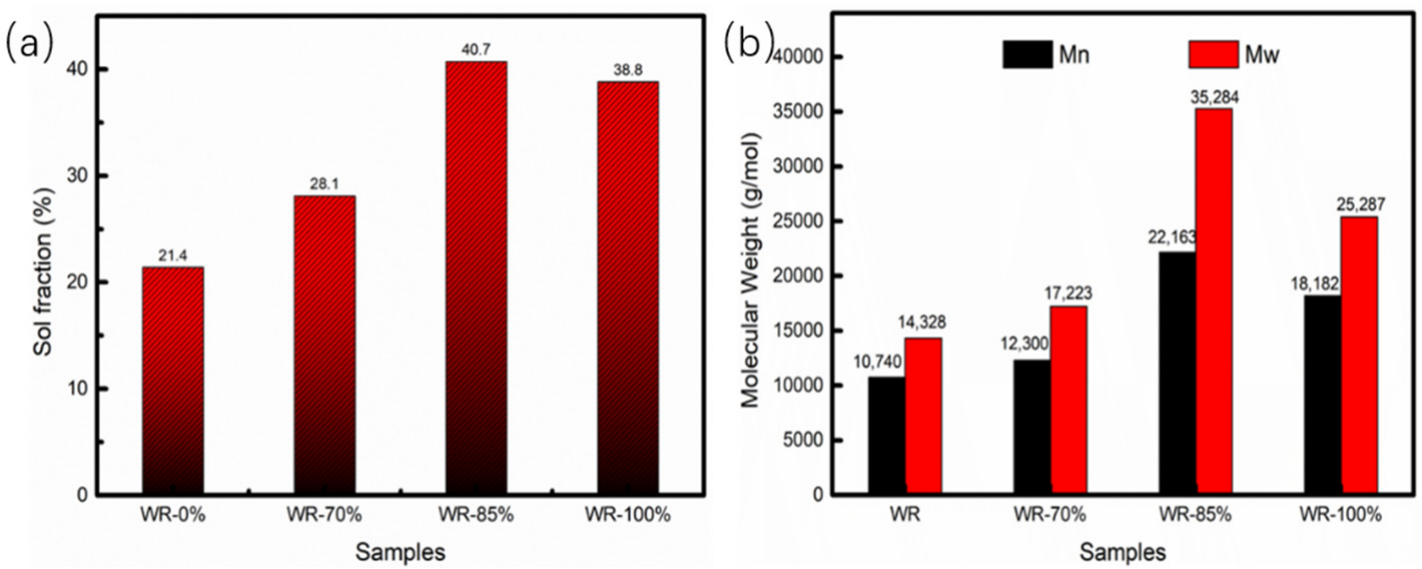
(**a**) Sol fraction of WR as a function of different filling rate. (**b**) Molecular weight of WR at different filing rate.

The sol fraction of WR and its molecular weight are shown in Figure 11a. Obviously, the pressure has a significant effect on the sol fraction of WR, and the sol fraction related to the degree of decrosslinking of samples changes significantly with the increase of filling rate. From the original WR material to 85% filling rate, the sol fraction increased from 21.4% to 40.7%, indicating that the crosslinking structure of WR is significantly damaged with the increase of filling rate. However, the sol fraction decreased from 85% to 100%, indicating that WR may be re-crosslinked.

GPC measurement of the sol samples revealed the changes of crosslinking structure and main chain of samples during the thermo-mechanical grinding process. The result is shown in Figure 11b. Compared with original WR, the Mw and Mn of the WR after grinding increased, owing to the decrosslinking of WR in the process of grinding. In addition, the Mw and Mn of WR are the largest at filling rate of 85% compared with the other two conditions, indicating that a lot of soluble rubber molecules with longer chain existed in the soluble components of WR, which leads to the increase of the molecular weight of WR, indicating that the degree of decrosslinking is the highest. As for the decrease of sol fraction at the filling rate of 100%, it should be due to the fracture of the main chain. Therefore, it can be concluded that suitable fill rate can effectively destroy the crosslinking structure of WR. In this study, the best decrosslinking effect is shown when the filling rate is 85%.

#### 3.3.5. Analysis of DSC

**Figure 12:**
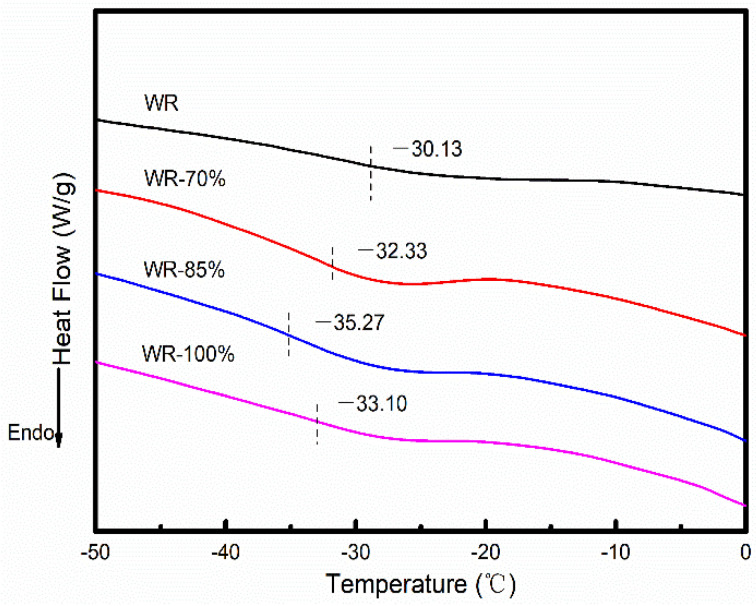
DSC curves of WR at different filling rate.

DSC curves are also applied to consider the T_g_ changes of WR at different filling rates and untreated rubber, as shown in Figure 12 and Table S4. Clearly, the T_g_ and χ_im_ values of WR have the largest change at the filling rate of 85%. Combining the results from sol fraction and molecular weight test, we can conclude that the cross-linked structures of WR were obviously destroyed after grinding.

### 3.4. Mechanism Discussion

**Figure 13:**
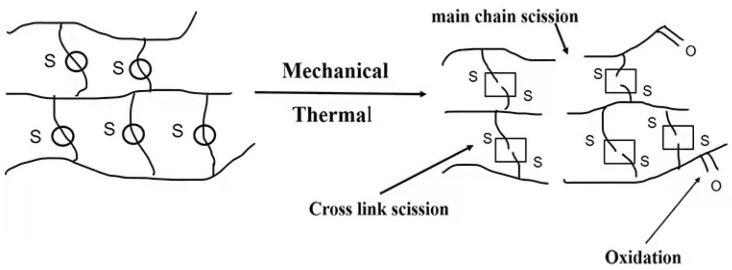
Fracture mechanism of WR during thermo-mechanical grinding.

Sustainable development of rubber-recycling techniques aims at reusing WR as close as possible to its original form. The type of recycling method is called decrosslinking and reclamation [15]. Fracture mechanism of WR is shown in Figure 13. As for chemically cross-linked rubbers, the chemical bonding energy of sulfur−sulfur bonds (S-S, 270 kJ/mol) is much weaker than that of carbon−sulfur bonds (C-S, 310 kJ/mol) or carbon−carbon bonds (C-C, 370 kJ/mol), so the S-S bonds in rubber are more susceptible to breakage. Therefore, thermal-mechanical grinding has the potential to be applied to decrosslinking of WR. It has been recognized that the unsaturated double bonds of the main chains combine with oxygen, which can cause a different degree of oxidative degradation under the action of heat [31]. The higher the temperature, the more susceptible it is to oxidative degradation. However, the temperature higher than 100 °C is more prone to serious oxidative degradation, resulting in a large amount of VOCs pollution. In the case of chemical bonds by forces, a basic understanding of the cleavage of crosslinking bonds under different filling rate is as follows, at extremely higher pressure induced by filling and kneading in the reactor, most of the rubber molecules may be fully elongated to their limited extensibility. The bonds having lower elastic constant (the S-S bonds) may become more extended in comparison with bonds having higher elastic constant (C-C bonds). The elastic energy induced by high pressure with a reasonable temperature, may be particularly effective on the S-S bonds, causing the selective breakage of crosslinking bonds along with lower energy consumption and environmentally friendly process in comparison with conventional recycled techniques [37]. This technique is also proved to have a good re-vulcanizing performance in the next part, which can be partly utilized to take the place of pure rubber.

### 3.5. RWR/SBR Vulcanizing Performance

**Figure 14:**
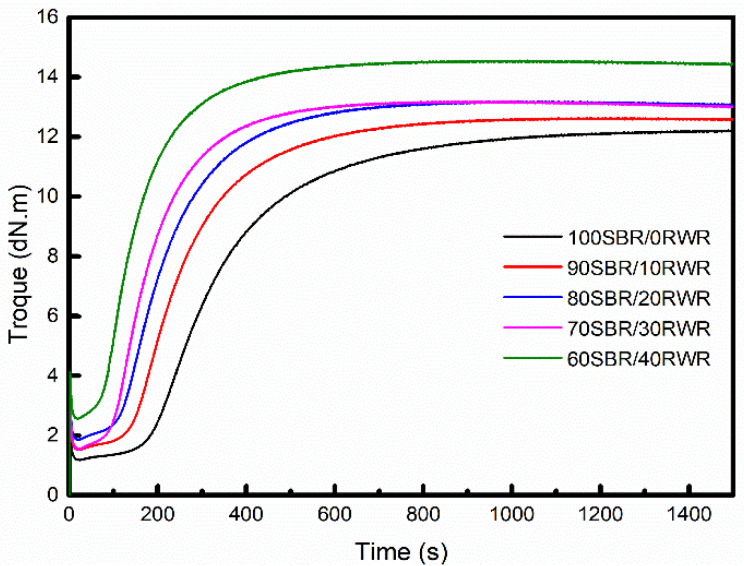
Vulcanization curves of RWR/SBR composites as a function of RWR content.

Application of different parts of rubber powder and fixed amount of carbon black to styrene-butadiene rubber, as well as the vulcanization and physical and mechanical properties of RWR/SBR composites were studied. The vulcanization curves and parameters of the RWR/SBR are shown in Figure 14 and Table 7, respectively. The re-vulcanization characteristics, on the whole, show a similar trend and ΔM values as pure SBR. With the increase of RWR, the optimum curing time (T_90_) of RWR/SBR was reduced from more than ten minutes to about five minutes, respectively. Because the RWR had a high-temperature crosslinking activating effect on the composites, therefore, both the Scorch time (T_S1_) and T_90_ were shortened [33]. Besides, the minimum torque (M_L_) and maximum torque (M_H_) of RWR/SBR increased with the increase of the fraction of RWR, which is related to the increase of system viscosity due to the residual cross-linked material in the RWR. The obvious reason for the increase of minimum torque is that the cross-linked RWR does not flow easily to the matrix, resulting in a higher viscosity of the mixture. The increase of maximum torque depends on the content of RWR because residual accelerators in RWR promote the increase of the degree of crosslinking of the composites [38].

### 3.6. Mechanical Properties of RWR/SBR

**Figure 15:**
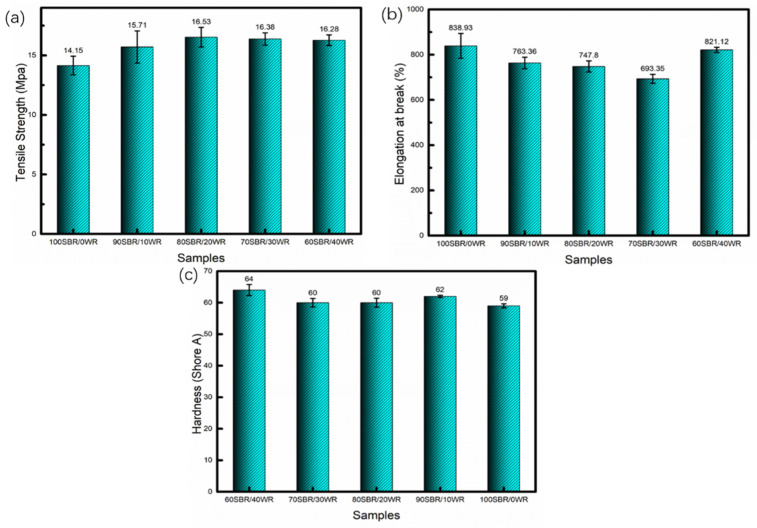
Mechanical properties of RWR/SBR composites as a function of RWR con-tent. (**a**) Tensile strength; (**b**) Elongation at break; (**c**) Hardness.

The mechanical properties of the RWR/SBR composites vulcanized at 160 °C are shown in Figure 15. Compared with SBR without RWR, the tensile strength slightly increases with the addition of RWR. The maximum value is 16.53 MPa with the addition of 20 wt% RWR, which may be due to the crosslinking activation of the sulfur in the vulcanization process, thus, improving the rubber crosslink density. This increase reflects the reinforcing effect of a filler particle, combined with a strong surface interaction with the SBR chains [39]. The decrease of tensile properties of RWR/SBR can be related to a certain degree of degradation of the rubber powder under the action of mechanical force. The value of elongation at break (Eb) shows a slight drop with further addition of RWR. The decrease in elongation at break of vulcanizates containing RWR indicates hindrance by RWR of deformability of the SBR matrix. The hardness value displays the same trend as tensile strength. These results can be attributed to the surface activation of the rubber powder caused by the thermal and high shear of grinding processing, which is beneficial for enhancing the interfacial bonding force between the RWR and the SBR matrix.

### 3.7. Micromorphology Analysis of RWR/SBR

**Figure 16:**
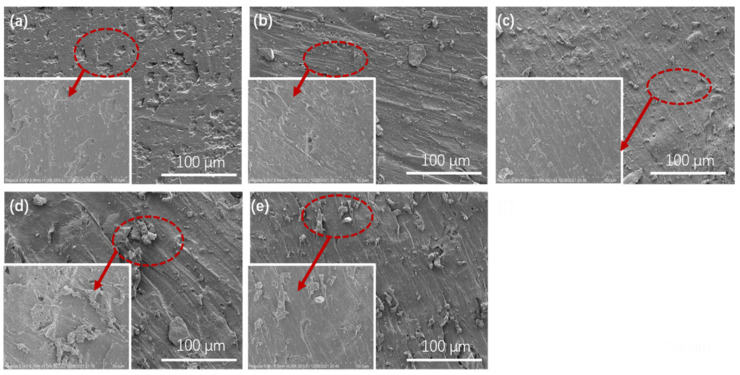
SEM images of RWR/SBR composites: (**a**) 100SBR/0RWR; (**b**) 90SBR/10RWR; (**c**) 80SBR/20RWR; (**d**) 70SBR/30RWR; and (**e**) 60SBR/40RWR.

Figure 16 shows the SEM image of the RWR/SBR composites, and the red circle in the picture represents the enlarged view of area in the RWR/SBR composites. Comparing Figure 16a with Figure 16b,c, it can be known that the addition of RWR significantly reduces the pitting on the surface of RWR/SBR composites, which suggests that the surface of the RWR was less exposed, and the dispersion was more uniform. The surface structure of RWR was changed and the bonding interface between the RWR and SBR matrix was improved under the dual action of mechanical force and additives. As for Figure 16d,e, the section protrusions and pits of the composite were significantly numerous, and the rubber powder obviously agglomerated and was exposed on the surface of the composite. This also contributes to the decrease of mechanical properties.

## 4. Conclusions

In this study, the influence of different temperatures and filling rates on the structure and properties of the waste rubber from the shoe industry was investigated by means of thermo-mechanical grinding technology. The results show that the temperature and filling rate have important effects on the physical and chemical structure of WR. The thermo-mechanical method could reduce the particle size of WR. Increasing the grinding temperature causes a decrease in the sample energy consumption and Tg, while sol fraction increases. While with the increase of filling rate, the sample energy consumption and sol fraction increase and the particle size decreases. Obviously, the main chain fracture was destroyed by high grinding temperature and filling rate. Obviously, the oxidation reaction occurs during the thermal-mechanical grinding. The particle size distribution of WR after being ground can be described by Rosin’s equation. The best effect of thermal mechanical grinding is the temperature of 80 °C with filling rate of 85%. The results of sol fraction, GPC and DSC show that the crosslinking fracture and main chain fracture were induced by thermal mechanical grinding, which destroys the crosslinking network of WR, resulting in the increase of sol fraction and molecular weight. The re-vulcanization characteristics, on the whole, show a similar trend and ΔM values as pure SBR. However, the increase of M_H_ depends on the content of RWR because residual accelerators in RWR promote the increase of the degree of crosslinking of the composites. The RWR with a high reclamation degree shows good dispersibility and reinforcement in SBR. Adding 10−40 phr RWR to SBR had no adverse effect on the mechanical properties; instead, it improves the mechanical properties of SBR. These results demonstrate that thermo-mechanical grinding under suitable condition is a cost-effective, reliable, and environmentally friendly method to recycled rubber, without any additional materials or chemical, providing some guidelines in the upgrading of waste rubber in the shoe industry.

## Figures and Tables

**Table 1:** Formulation of SBR/RWR composites (phr).

SBR	RWR	CB	Zinc Oxide	Stearic Acid	CBS	Sulfur
100	0	30	5	2	1.5	2
90	10	30	5	2	1.5	2
80	20	30	5	2	1.5	2
70	30	30	5	2	1.5	2
60	40	30	5	2	1.5	2
50	50	30	5	2	1.5	2

**Table 2:** Energy consumption of materials at different temperatures.

Samples	Temperature (°C)	E_M_ (J)
WR-30 °C	30	44,315.5
WR-80 °C	80	35,165.3
WR-140 °C	140	18,414.4

**Table 3:** Rosin–Rammler–Bennet parameters of WR grinding process at different temperatures.

	30 °C	80 °C	140 °C
*D^e^*	843.65	829.20	869.44
*n*	4.13	2.93	3.54
R^2^	99.80	99.94	99.85

**Table 4:** Elemental analysis of WR and reclaimed WR obtained by XPS spectra.

Element	S2p	C1s	O1s
WR (wt%)	0.57	97.17	2.26
WR-80 (wt%)	0.69	86.69	12.62
WR-140 (wt%)	0.39	90.4	9.21

**Table 5:** Energy consumption of materials at different filling rates.

Samples	Percentage (%)	E_M_ (J)
WR-75%	70	18,006.8
WR-85%	85	35,165.3
WR-100%	100	69,749.4

**Table 6:** Particle size distribution of WR from different filling rates.

	70%	85%	100%
*D^e^*	639.82	625.48	593.48
*n*	3.49	3.59	2.64
R^2^	97.14	99.11	97.12

**Table 7:** Vulcanization parameters of RWR/SBR at 160 °C.

RWR/Phr	t_90_/min	t_s1_/min	t_s2_/min	ML/dN·m	M_H_/dN·m	ΔM/dN·m
0	10:50	3:11	3:39	1.18	12.21	11.03
10	8:10	2:27	2:49	1.52	12.63	11.11
20	7:09	1:59	2:20	1.86	13.18	11.32
30	5:55	1:41	1:57	2.20	13.84	11.64
40	5:24	1:19	1:34	2.56	14.55	11.99

## Data Availability

The data presented in this study are available on request from the corresponding author.

## Supplementary Materials

The following supporting information can be downloaded at: https://www.mdpi.com/article/10.3390/polym14051057/s1. Figure S1: Analysis of the WR. Figure S2: Torque (M) as a function of the grinding time between WR and reclaimed WR at room temperature. Table S1: Particle size distribution of WR from different temperatures. Table S2: Variation in glass transition temperature (T_g_), corrected enthalpy ΔCp and weight fraction (χ_im_) of the immobilized polymer layer (ΔH_m_) of WR at different grinding temperature. Table S3: Particle size distribution of WR from different temperatures. Table S4: Variation in glass transition temperature (T_g_), corrected enthalpy ΔCp and weight fraction (χ_im_) of the immobilized polymer layer (ΔH_m_) of WR at different filling rate.
