# Supplementary material for: Selective Decomposition of Waste Rubber from the Shoe Industry by the Combination of Thermal Process and Mechanical Grinding
Source: Polymers (Basel). 2022 Mar 7;14(5):1057. doi: 10.3390/polym14051057 (PMC8914881; doi:10.3390/polym14051057)
Supplement: Supplementary file 1 [file polymers-14-01057-s001.zip › polymers-1552269-supplementary.pdf]

polymers

MDPI

Supplementary

Selective Decomposition of Waste Rubber from the Shoe Industry by the Combination of Thermal Process and Mechanical Grinding

Qiao Xiao [1,2], Changlin Cao [2,3,4,*], Liren Xiao [1,2,*], Longshan Bai [1,2], Huibin Cheng [2,3,4], Dandan Lei [1,2], Xiaoli Sun [2,3,4], Lingxing Zeng [2,3,4], Baoquan Huang [2,3,4], Qingrong Qian [2,3,4] and Qinghua Chen [2]

[1] College of Chemistry and Materials, Fujian Normal University, Fuzhou 350007, China; qsz20191121@student.fjnu.edu.cn (Q.X.); qsz20191117@student.fjnu.edu.cn (L.B.); qsx20190767@student.fjnu.edu.cn (D.L.);
[2] Engineering Research Center of Polymer Green Recycling of Ministry of Education, Fujian Normal University, Fuzhou 350007, China; qbx20190099@yjs.fjnu.edu.cn (H.C.); sunxiaoli@fjnu.edu.cn (X.S.); zenglingxing@fjnu.edu.cn (L.Z.); qbh811@sina.com (B.H.); qrqian@fjnu.edu.cn (Q.Q.); cqhuar@fjnu.edu.cn (Q.C.)
[3] College of Environmental Science and Engineering, Fujian Normal University, Fuzhou 350007, China
[4] Fujian Key Laboratory of Pollution Control & Resource Reuse, Fujian Normal University, Fuzhou 350007, China
* Correspondence: caochlin3@fjnu.edu.cn (C.C.) and xlr1966@fjnu.edu.cn (L.X.)

In order to understand the composition and filler content of WR, the structural analysis of WR is shown in Figure S1 and Table S1. The appearance of WR is shown in Figure S1a. As shown in Figure S1b, the macromolecular structure of the WR was investigated using FTIR spectroscopy. The absorbance peak at 3428 cm^{-1} is assigned to the -OH groups, the new peaks observed at 2917 and 2851 cm^{-1} is related to the stretching vibration of asymmetric and symmetric -CH, respectively. The main bands of calcium carbonate is attributed to the elongation of CO_3^{2-} group at approximately 1456 cm^{-1}. The bending vibration peaks of the CH_2 group at 1390 cm^{-1}. The peak at 1733 cm^{-1} associated with C=O. Moreover, 1633 cm^{-1} is attributed to C=C absorption. The tensile vibration of O-Si-O is 1105 cm^{-1}, the peak at 970cm^{-1} shows the -CH=CH- in-plane deformation of trans-1,4 polybutadiene for butadiene rubber (BR). Therefore, determination the components of WR contains SBR and BR, and the fillers contains $CaCO_3$ and SiO_2 (white carbon black).

Raman scattering spectroscopy was also used for characterization. The presence of impurity and addition of crosslinking agents, fillers and other rubber ingredients into rubber are the main reasons of fluorescence parasite signals in Raman scattering spectrum. Consequences follow from Figure S1c, both symmetric and asymmetric–CH_2 and -CH_3 stretching vibrations typically appear in the 2800-3000 cm^{-1} region. Apparently, C=C stretching vibration of WR is observed at 1655 cm^{-1}, -CH_2 in-plane deformation and twisting are observed at 1444 cm^{-1} and 1269 cm^{-1}. The peak appeared at 1002 cm^{-1} is corresponding symmetric ring breathing of C=C bonds. Obviously, FTIR spectroscopy and Raman spectroscopy, were concluded that the main component of WR is SBR/BR blend.

Figure S1d shows the TG result of WR, which presents three major weight loss events. The first stage at 200-350 °C is attribute to the decomposition of low molecular weight substances，such as additives, which is a weight loss (approximately 20%). The second stage in the range of 350-480 °C is associated with degradation of the rubber matrix(SBR/Brand), the weight loss of the second stage about 50%. The third stage at 480-670 °C is related to with the decomposition of calcium carbonate ($CaCO_3$), and there are about 5% of weight loss at this stage.

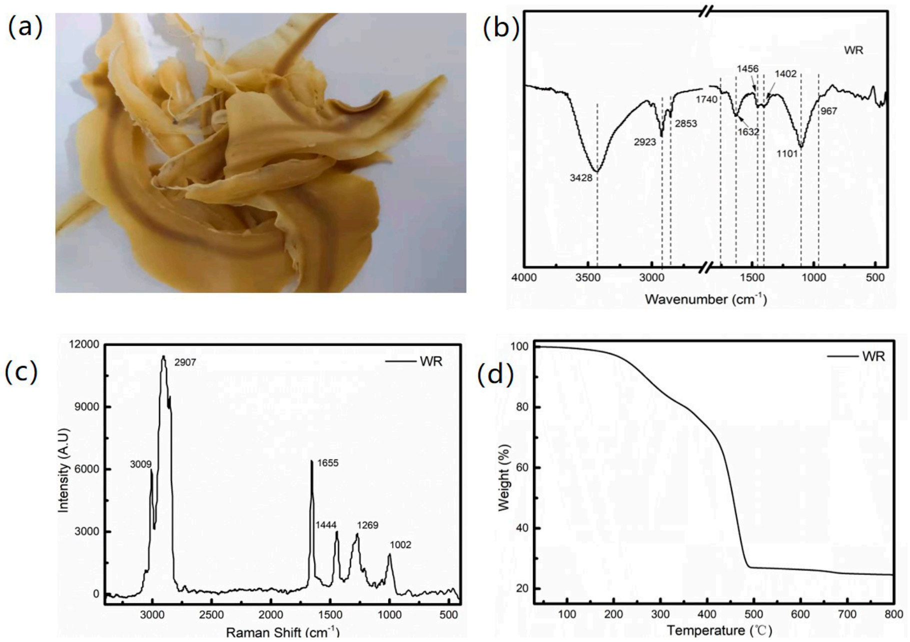

Figure S1. Analysis of the WR. (**a**) The appearance of the WR. (**b**) FTIR spectra of WR. (**c**) Raman spectra of WR. (**d**) TG analysis of WR.

The torque curves of WR and reclaimed WR at room temperature in Figure S2. Obviously, the reclaimed WR has a relatively lower torque as compared to WR, indicating lower viscosity.

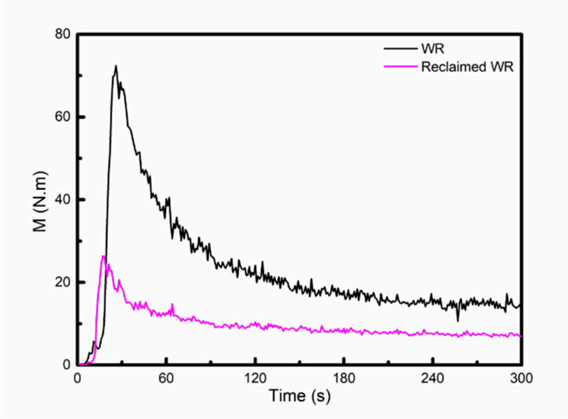

Figure S2. Torque (M) as a function of the grinding time between WR and reclaimed WR at room temperature.

Tables

Table S1. Particle size distribution of WR from different temperatures.

Samples	D_{10}	D_{50}	D_{90}
WR-30 °C	408.27 μm	607.64 μm	765.46 μm
WR-80 °C	259.68 μm	579.96 μm	760.41 μm
WR-140 °C	487.82 μm	772..30 μm	1119.56 μm

Table S2. Variation in glass transition temperature (Tg), corrected enthalpyΔCp and weight fraction (χ_{im}) of the immobilized polymer layer (ΔH_m) of WR at different grinding temperature.

Samples	T_g(°C)	ΔC_p (J^{-1}.g)	λim
WR	-30.12	0.126	-
WR-30 °C	-31.02	0.112	0.016
WR-80 °C	-35.27	0.087	0.044
WR-140 °C	-36.37	0.064	0.071

Table S3. Particle size distribution of WR from different temperatures.

Samples	D_{10}	D_{50}	D_{90}
WR-70%	146.75 μm	601.68 μm	766.45 μm
WR-85%	259.68 μm	579.96 μm	760.41 μm
WR-100%	125.48 μm	552.16 μm	755.01 μm

Table S4. Variation in glass transition temperature (Tg), corrected enthalpyΔCp and weight fraction (χ_{im}) of the immobilized polymer layer (ΔH_m) of WR at different filling rate.

Samples	T_g(°C)	ΔC_p (J^{-1}.g)	λim
WR	-30.12	0.126	-
WR-70%	-32.33	0.261	-0.15
WR-85	-35.27	0.087	0.044
WR-100%	-33.10	0.118	0.009
